# Ethno-entomotherapeutic and metabolite profiling of *Coridius chinensis* (Dallas), a traditional edible insect species of North-East India

**DOI:** 10.1038/s41598-024-57202-y

**Published:** 2024-03-19

**Authors:** Rajkumari Bonysana, Kabrambam Dasanta Singh, Wahengbam Deepanita Devi, Arunkumar Singh koijam, Kokho Kapesa, Jatin Kalita, Pulok Kumar Mukherjee, Yallappa Rajashekar

**Affiliations:** 1 Insect Bioresources Laboratory, Animal Resources Programme, Institute of Bioresources and Sustainable Development (IBSD, Department of Biotechnology, Govt. of India, Takyelpat, Imphal, Manipur 795001 India; 2 Centre for Infectious Disease, CSIR-North East Institute of Science and Technology, Jorhat, Assam India

**Keywords:** *Coridius chinensis*, Edible insects, Antioxidant activity, Anti-inflammatory activity, Traditional medicine, Entomology, Animal biotechnology

## Abstract

Edible insects possess several health enhancing properties and play an important role in human nutrition. *Coridius chinensis* is an edible insect that is considered food and claimed as traditional medicine. In the present study, nutritional contents, chemical composition, antioxidant, and anti-inflammatory properties of *C. chinensis* were analyzed. It was found that the insect sample contains 50.46% moisture, 44.65% protein, 4.45% carbohydrate, 39.42% crude fats, 3.53% ash and 576.11 (Kcal/100 g) energy. Our study highlighted the presence of a significant amount of phenol and flavonoids. The *C. chinensis* hydro-alcoholic extract showed high antioxidant property and anti-inflammatory activity. GCMS analysis identified 61 volatile compounds. LC–MS analysis of hydroalcoholic extract of *C. chinensis* revealed the presence of compounds such as etodolac glucuronide, morphine 3-glucuronide, ecgonine, ecgonine methyl ester, sufentanil, and palmitoyl ethanololamide. These findings suggest that *C. chinensis* species can be employed as a valuable food source with excellent therapeutic properties.

## Introduction

Food is the basic requirement of an organism to survive as it provides various nutrients, and numerous chemical compounds beneficial for growth and development of an organism^1^. Humans depend directly or indirectly on plants, fungi, and animals as food source. Among them insects form an integral part by providing essential nutrients to human^2,3^. Entomophagy or the consumption of insect as food by human is an age old, well-known practice in different parts of the world^4^. Edible insects undeniably play an important role in reducing the food insecurity as well as nutrients deficiency in many parts of the world especially in Asia, Africa, and Latin America^5,6^. A very important feature of edible insects is their nutraceutical and therapeutic properties, and this is attributed to the presence of secondary or primary metabolites^7^. The purpose of entomophagy differs from community to community depending on the insect’s availability, nutritional, medicinal value, and social belief as well as on local tradition and customs^8,9^.

**Figure 1:**
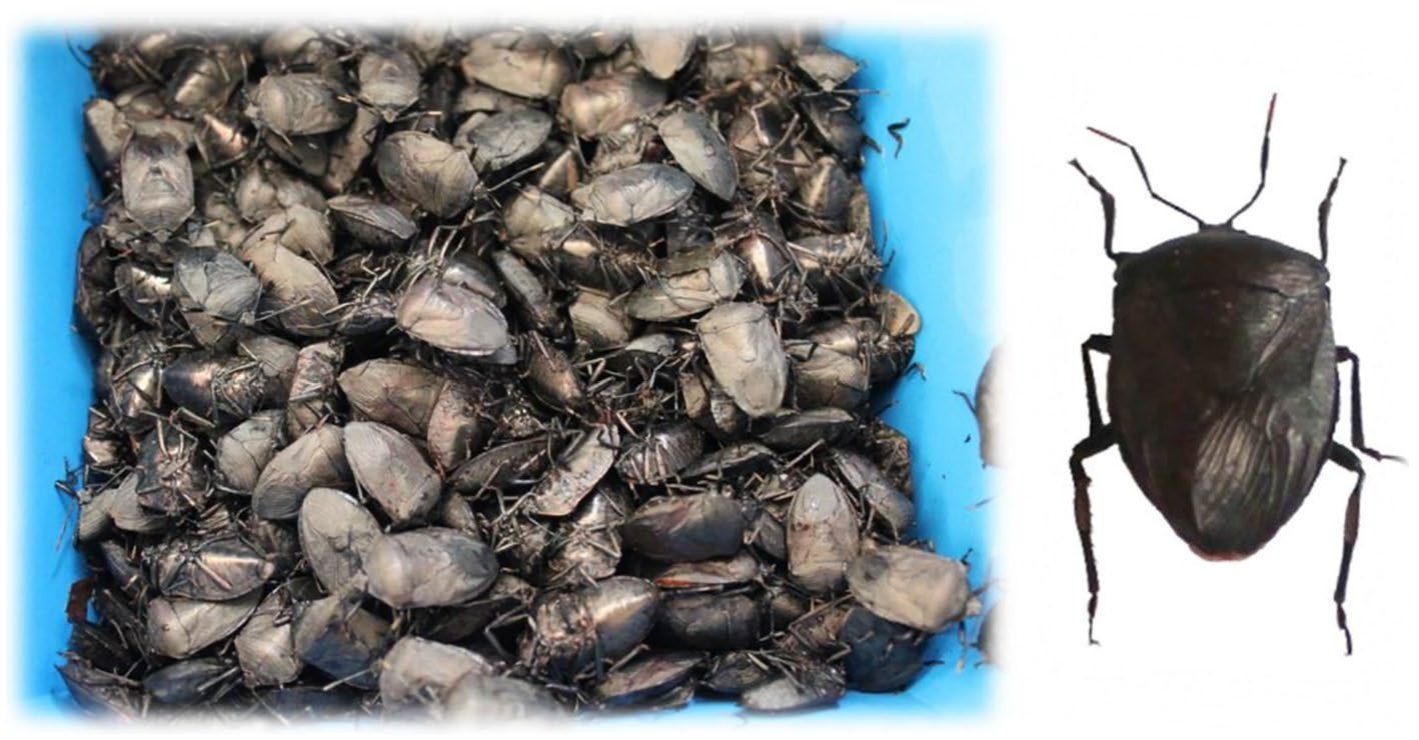
Pictorial representation of *Coridius chinensis.*

Since the beginning of the twentieth century, entomophagy has been well-known in North-East India, including Manipur, Nagaland, Arunachal Pradesh, and other places^3,9,10^. *Coridius chinensis* (Dallas), a stink bug species is one of the many insects consumed by people in Ukhrul district of Manipur and is locally known as “Lenghik” (Fig. 1). It belongs to the family Dinidoridae (Hemiptera). Generally, this bug is available during the onset of winter months under the climatic conditions of north-eastern India. Cultural and traditional practice of *C. chinensis* collection is generally done for non-commercial household consumption. It is eaten cooked, fried, or roasted, mostly for flavor. *C. chinensis* has been reported as an ethnomedicine for the treatment of joint pain by the local Tangkhul community of Ukhrul. It is also used as a traditional Chinese medicine, to relieve pain, jaundice, and to help those who have trouble breathing^11^. It has also been reported to have antioxidant properties, thus countering the free radicals and reactive oxygen species generated in physiological systems and adding another entomo-therapeutic aspect^12^.

In the present study, preliminary qualitative screening of primary and secondary metabolites, analysis of chemical composition, *in-vitro* antioxidant activity and anti-inflammatory activity from the crude extract of *C. chinensis* were studied. Liquid Chromatography Mass Spectrometry (LCMS) was employed to elucidate various metabolites in the range of 50–1300 m/z.

## Materials and methods

### Sample collection and preparation of crude extract

*C. chinensis* samples were collected from Ukhrul market, Ukhrul District of Manipur, India, at the co-ordinates N- 24°58′ 55′′ E- 94°13′ 34′′. The specimen was identified by the Insect Biosystematics and Conservation laboratory (Ashoka Trust for Research in Ecology and the Environment, Bengaluru, Karnataka). Adult insects were thoroughly cleaned under running water, dried at room temperature, and stored at − 20 °C until further experiments. Hydro-alcoholic extract was prepared by dissolving 30 g of finely powdered insects in 150 ml mixture of methanol and water (70:30, v/v) with intermittent shaking for five days. Extraction of fats was done from dried powdered insects using n-hexane subjected to the Soxhlet apparatus for 8–9 h at 40 °C. The hydro alcoholic and n-hexane extracts were filtered with Whatman paper No. 1, and concentrated under low pressure using rotary vacuum evaporator {Rotavapor R100 (Buchi) Switzerland} at 45 °C.

### Preliminary qualitative analysis of insect’s metabolites

#### Ninhydrin test

1 ml of each extract was taken in dry test tube. A few drops of 2% ninhydrin were poured in both the test tubes and kept in water bath for 5 min. Distilled water was used as control in place of extract in a separate tube. Observation was made for any development of color in the test tubes with blue or violet color indicating the presence of Ninhydrin Proteins (Amino acid/ proteins)^7^.

#### Salkowski’s test

In a mixture of 0.5 ml of chloroform and 1 ml concentrated H_2_S0_4_, another 1 ml of extract was gently added to form a layer. Terpenoids presence in the sample was confirmed if a reddish-brown coloration develops in the interface of the layer^13^.

#### Keller–Kilian’s test

In 1 ml of extract, 0.5 ml of glacial acetic acid was added, followed by a few drops of FeCl_3_. 1 ml of concentrated H_2_S0_4_ was carefully added into the mixture. The presence of cardiac glycosides was determined by formation of a brown ring at the interface^13^.

#### Ferric chloride test

This test was done to confirm the presence of tannins in a given sample**.** Here, 1 ml of each extract was diluted with 1 ml distilled water, followed by the addition of 2 drops of ferric chloride. A transient greenish to black color indicates the presence of tannins^14^.

#### Saponin

In 1 ml of extract, 3 ml of dist. H_2_O was added and boiled in water bath. It was then filtered and shaken vigorously for a stable persistent froth, and observed for the formation of emulsion which confirms the presence of Saponin^15^.

#### Mayer’s test

The presence of alkaloids was tested by mixing the extract and Mayer’s reagent in the ratio of 1:2 and observing for the formation of any cream-colored precipitate^13^.

### Proximate composition of *C. chinensis*

The method given by Association of Official Analytical Collaboration (AOAC)-934 was followed to estimate the moisture content of the sample. The initial weight of the insects was taken and then kept for drying in an incubator at 60 °C. After drying completely, the final weight was taken and the difference between initial and final weight represents the moisture content (expressed in percentage). The hydro-alcoholic extract was used to study the proximate composition of *C. chinensis.* Total protein estimation was conducted by following the method of Lowry assay using bovine serum albumin as standard, with slight modification^16^. Total Carbohydrate estimation was done by Anthrone method using Glucose as standard. However, the crude fat percentage was obtained following AOAC guidelines from n-hexane extract^17^.

### Determination of total phenol and flavonoids content

The total phenol content was estimated by using Folin–Ciocalteu’s method with slight modification^18^. The reaction was measured by spectrophotometer at 725 nm. 1 mg/ml hydro-alcoholic extract was mixed with Folin-Ciocalteu’s phenol reagent and kept for 5 min at room temperature. Into the mixture, 7.5% Na_2_CO_3_ solution was added, followed by distilled water. For the reaction to occur, which is indicated by the formation of blue colour, the mixture was incubated at room temperature for 90 min in the dark. Gallic acid was used as a standard. The total phenolic content was determined from extrapolation of a calibration curve, plotted using gallic acid standard. The estimation of the phenolic compounds was carried out in triplicate.

Determination of total flavonoid content in the hydro-alcoholic extract was done following the method of Park et al., (2008)^19^. The standard curve for total flavonoids was extrapolated using Quercetin standard solution. Both the extract and standard solution were mixed properly, and the absorbance was read against the reagent blank at 506 nm. The total flavonoid content in the extract was expressed as mg of quercetin equivalent/g of extract.

### In-vitro evaluation of antioxidant activity of *C. chinensis*

The antioxidant property of the hydro-alcoholic extract of *C. chinensis* was studied *in-vitro* following different methods of biochemical assays such as superoxide dismutase (SOD), Glutathione-S-transferase (GST), 1,1-diphenyl-2-picrylhydrazyl (DPPH) scavenging activity and 2,2′-azino-bis (3-ethylbenzothiazoline-6-sulfonic acid) (ABTS) radical scavenging property. All the experiments were performed in triplicates.

### SOD assay

The free radical scavenging activities of sample extract was measured by Superoxide dismutase assay. As described by Marklund and Marklund (1974)^20^, superoxide dismutase (SOD) activity was measured using pyrogallol (2 mM) auto-oxidation, with slight modification. The reaction mixture contained the whole insect homogenate at different concentrations of 2.5, 5, 7.5, 10 and 12.5 µg/µl, 0.1 M tris buffer (pH 8.2) and pyrogallol. Distilled water was used as control. The substrate was added at last, initiating the reaction and the absorbance was read at 420 nm immediately. The inhibition of pyrogallol autoxidation by SOD activity of the extracts is expressed in percentage.

### Glutathione-S-transferase

Glutathione-S-transferase (GST) activity was measured by following the method of Warholm et al., (1985)^21^ with 1-Chloro-2,4-dinitrobenzene (CDNB) as the substrate. 10 μL samples at concentration 2.5, 5, 7.5,10, and 12.5 µg/µl were added in individual reaction mixtures containing 0.1 M phosphate buffer, and 20 mM GSH. Distilled water was used as control. The reaction was started by adding 30 mM CDNB at 37 °C and the change in absorbance was observed using a spectrophotometer at wavelength 344 nm. The enzyme activity was expressed as µmole CDNB conjugate/min/mg protein.

### DPPH radical scavenging activity

1,1-diphenyl-2-picrylhydrazyl (DPPH) radical scavenging properties of the sample extracts was measured by following the methods described by Yamaguchi et al. (1998)^22^ with slight modification. Briefly, 1 ml of 0.1 mM DPPH solution in 95% ethanol was treated with different concentrations (2.5, 5, 7.5,10, and 12.5 µg/µl) of the dried sample. They were properly mixed and incubated at room temperature for 30 min and the absorbance was read at 517 nm. Ascorbic acid at different concentrations of 0.25, 0.5, 0.75, 1.0, and 1.25 µg/µl, was used as a standard. The radical scavenging activity was calculated using the following formula.

$${\text{Scavenging effect }}\left( \% \right) \, = \, \left[ {{1} - \frac{{{\text{Absorbance of the Sample }}\left( {517{\text{nm}}} \right){ }}}{{{\text{Absorbance of the Control }}\left( {517{\text{nm}}} \right)}}{ } \times { }100} \right]$$

### 2,2′-azino-bis (3-ethylbenzothiazoline-6-sulfonic acid) (ABTS) assay

ABTS Assay was determined according to Re et al. (1999)^23^ with some modifications. A mixture of 7 mM ABTS and 140 mM ammonium persulfate was prepared and allowed to stand overnight at room temperature and diluted with ethanol to obtain an absorbance of 0.70 (± 0.02) at 734 nm. In a 96-well microplate, 20 µL of the sample at different concentrations of 2.5, 5, 7.5,10, and 12.5 µg/µl each and 180 µL of ABTS solution were mixed and incubated for 30 min at room temperature. Trolox at different concentrations of 0.25, 0.5, 0.75, 1.0, and 1.25 µg/µl, was used as a standard. Absorbance was measured at 734 nm and the percentage scavenging activity was calculated according to the following formula.

$${\text{Scavenging activity }}\left( \% \right) \, = \frac{{{\text{Absorbance of control}} - {\text{Absorbance of sample}}}}{{\text{Absorbance of control }}} \times 100$$

### In vitro anti-inflammatory bioassay (protein denaturation assay)

The bioassay was performed following Banerjee’s et al. (2014)^24^ with slight modification. The individual reaction mixtures with final volume of 4 ml consisted of 0.5 ml of 1% bovine albumin, 1 ml phosphate buffered saline (pH 6.4) and test extracts to obtain a final concentration of 1000, 1400, 1800, 2200, 2600 and 3000 μg/ml. Distilled water was used to make up the final volume. The well mixed reaction mixture was incubated in a water bath at 37 °C for 15 min, and then exposed to a high temperature of 70 °C for 5 min. Double distilled water was used as control. It was then allowed to cool, and the absorbance was read at 660 nm in a spectrophotometer. Diclofenac at the final concentrations of 20, 40, 60, 80, 100, 150, and 200 μg/ml were used as reference. The experiment was performed three consecutive times, and the mean absorbance was taken to calculate the percentage inhibition of protein using the following formula.

$${\text{Percentage Inhibition }} = \frac{{{\text{Absorbance of control}} - {\text{Absorbance of test}}}}{{\text{Absorbance of Control}}} \times 100.$$

### Gas chromatography-mass spectrometry (GC–MS) analysis

GCMS analysis was performed to determine the chemical compositions of hydro-alcoholic extract of *C. chinensis*. The volatile constituents of the extract were analyzed by using Thermo Scientific (Trace 1300 Gas Chromatograph & TSQ 8000 DUO Mass Spectrometry). The instrument specification includes a Quadrupole detector analyses attached to TG-5MS and a silica capillary column of (30 m × 0.25 mm inner diameter and 0.25 μm film thickness). The GC–MS detection was performed with ionization energy optimized at 70 eV. While the injector and mass transfer lines temperature were set at 250 °C and 280 °C respectively. Helium (carrier gas) at a flow rate of 1 ml/min was used as a mobile phase that will bring down the solute up to the column and the injection volume of each sample was 0.5 μl (1:100 samples in methanol). The column was initially exposed to 40 °C for 1 min and then gradually raised to 250 °C at a rate of 5 °C/min heating ramp and maintained at 250 °C for 20 min. The compounds were identified by comparing the mass spectra with those of the National Institute of Standards and Technology (NIST) GC–MS Libraries 2017, respectively.

### Liquid chromatography mass spectrometry (LC–MS) analysis

The hydroalcoholic extract was suspended in methanol to obtain a concentration of 1 µg/ml followed by filtration through 0.20 μm membranes. LC–MS was performed using an Agilent LC-QTOF 6546 system with a C18 column of the dimensions 2.1 × 50 mm, 1.8 μm (Agilent ZORBAX Eclipse Plus). Mobile phases were constituted by 0.1% formic acid in water (A) and acetonitrile (B) as eluents in gradient mode. The injected volume was 5.0 μL, and the column temperature was maintained at 35 °C. The column elution was done at a constant flow rate of 0.200 μL/min throughout a linear gradient as follows: 2% (B) at 0–2 min, 5% (B) at 2–5 min, 35% (B) at 5–8 min, 50% (B) at 8–15 min, 75% (B) at 15–20 min, 5% (B) at 20 min. The QTOF mass spectrometer has Dual AJS ESI as ion source. The analysis parameters were set with positive ion polarity mode and a spectral acquisition of mass ranging from *m/z* 100–1300 with scan rate of 2 spectra.

The MS data analysis for compound identification was executed using Mass Hunter vB.0 8.00 software with integrated library viz. Metlin_Metabolites _AM_PCDL was used with Molecular feature extraction. The list of compounds was then filtered by limiting the score to 75% and above only.

### Statistical analysis

The data were represented as mean ± standard error (SEM). One-way ANOVA, followed by Tukey’s multiple range test was used to analyze significant differences at *p*-value < 0.05, using GraphPad prism 8.4.0.

## Results

### Qualitative analysis of insect’s metabolites

The presence of various primary and secondary metabolites in both *C. chinensis* hexane and hydro-alcoholic extracts were studied following their respective standard protocols. It showed the presence of metabolites such as flavonoids, terpenoids, alkaloids, reducing sugars and proteins etc. in the hydro-alcoholic extract. Whereas, in hexane extract, only reducing sugars and saponins were found to be present. The details of the results are presented in Table 1.

**Table 1:** Preliminary qualitative test for the presence of insect’s metabolites.

S.N	Test compound	Extract test	Hexane extract	Hydro-alcoholic extract
1	Protein	Ninhydrin test	−ve	+ ve
2	Reducing sugar	Benedict’s test	+ ve	+ ve
3	Alkaloids	Meyer’s test	−ve	+ ve
4	Terpenoids	Salkowski’s test	−ve	+ ve
5	Cardiac glycosides	Keller-Kilian’s test	−ve	+ ve
6	Saponin	Saponin	+ ve	+ ve
7	Flavonoids	Alkaline test for flavonoids	−ve	+ ve

+ ve indicates present, −ve indicates absent.

### Proximate composition of *C. chinensis*

Parameters such as moisture, crude proteins, carbohydrates, fats and ash content were analyzed for *C. chinensis*. The percentage content of moisture, proteins, carbohydrates, and crude fats were 50.4%, 22.12%, 2.20%, and 19.53% respectively (Table 2).

**Table 2:** Proximate composition of *C. chinensis.*

Nutrients	Percentage composition
Dry weight	49.175 ± 0.63
Protein	44.65 ± 0.018
Carbohydrate	4.45 ± 0.23
Crude Fats	39.42 ± 0.65
Ash	3.53 ± 0.06
Energy (Kcal/100 g)	576.11 ± 19.46

Moisture content based on fresh weight of *C. chinensis* is 50.45 ± 0.667.

Values are expressed as mean ± sem, n = 4.

### Determination of total phenols and flavonoids

**Figure 2:**
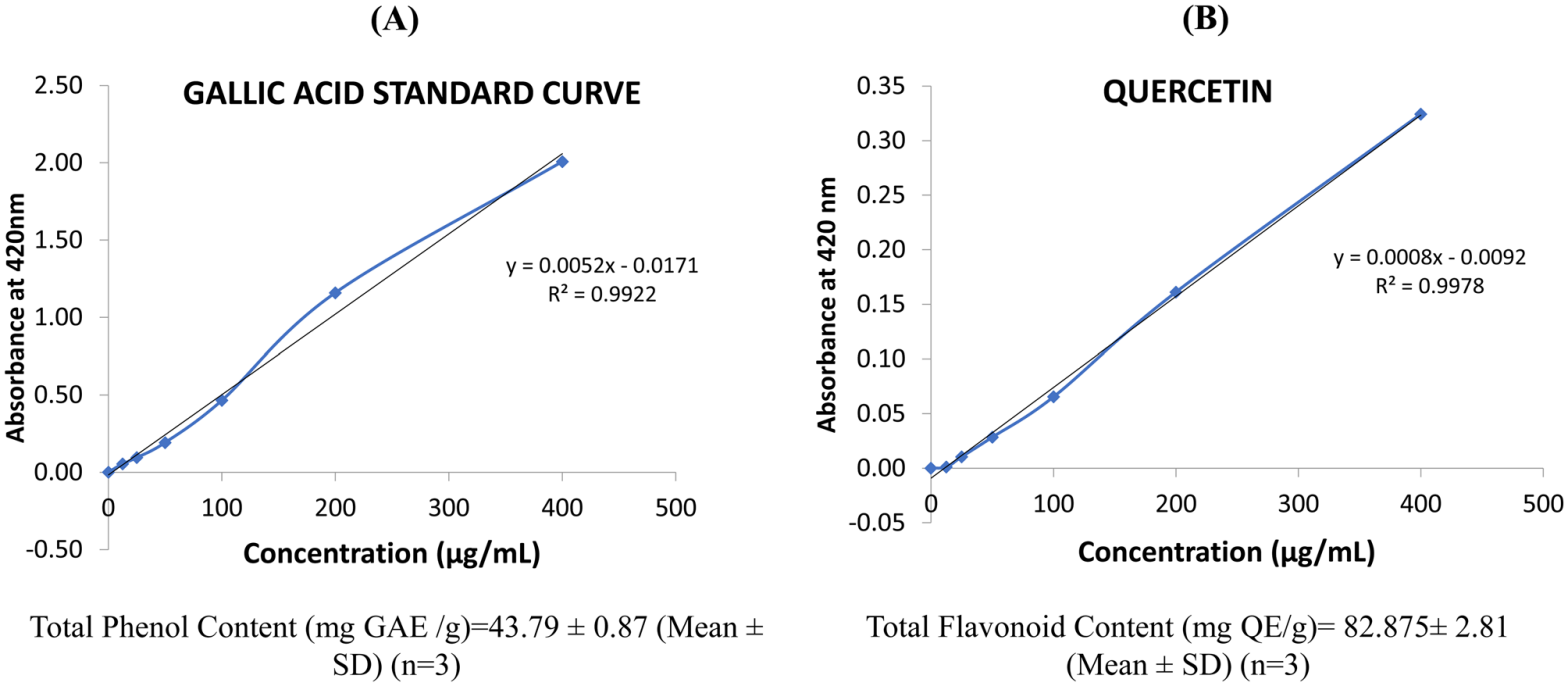
(**A**) The total phenolic content was determined from extrapolation of a calibration curve, plotted using gallic acid standard. (**B**) The standard curve for total flavonoids was made using Quercitin standard solution with the same concentration used in the phenol determination.

The quantitative determination of the total phenols and flavonoids was performed from the hydro alcoholic extract of *C. chinensis*, using gallic acid and quercetin as respective standard. Relatively good amounts of phenols (43.79 ± 0.87 mg GAE /g) and flavonoids 82.875 ± 2.81 mg QE/g) were present in *C. chinensis* (Fig. 2A,B).

### Antioxidant assay

**Figure 3:**
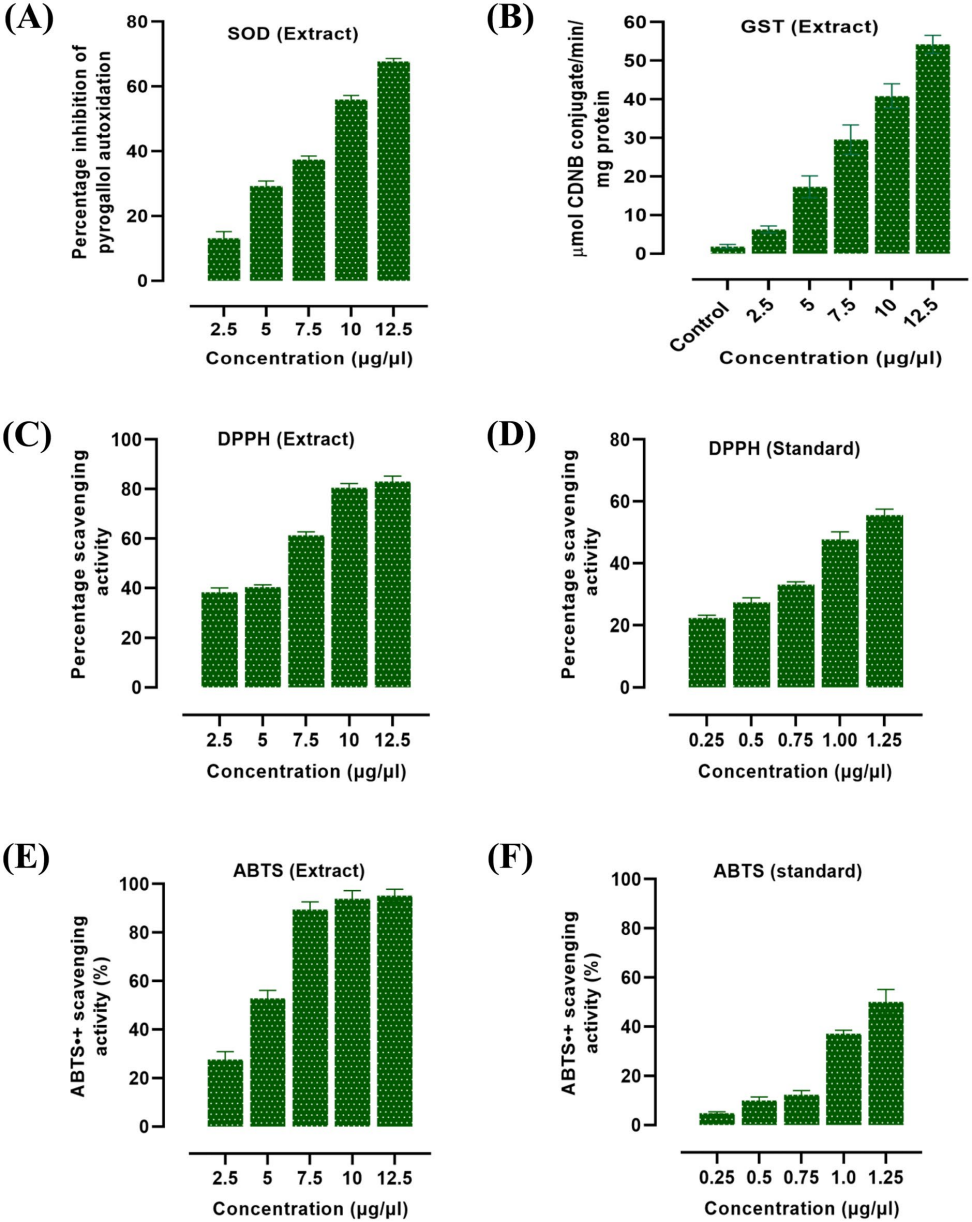
In-vitro antioxidant activities of different solvent extracts of *C. chinensis.* (A1, A2, and A3) represent the SOD activities of hexane extract, ethyl acetate extract and methanol extract respectively. (B1, B2, and B3) represent GST activities of hexane extract, ethyl acetate extract and methanol extract respectively. (C1, C2, C3, and C4) represent the DPPH free radical scavenging activities of hexane extract, ethyl acetate extract, methanol extract, and standard (Ascorbic acid) respectively. (D) Represents ABTS• + scavenging activity of hexane extract, ethyl acetate extract and methanol extract respectively.

The study demonstrated significant antioxidant activity by performing different assays namely SOD, DPPH, and GST in the hydro-alcoholic extract of *C. chinensis*. Superoxide dismutase (SOD) is one of the key enzymes of insect antioxidant system^25^. The importance of SOD in therapeutic potential and physiological have already been mentioned in several reports. Here, we evaluated the SOD activity measuring the inhibition of pyrogallol autoxidation. The percentage inhibition of pyrogallol autoxidation due to SOD activity of the extract was highest at dose of 12.5 µg/µl with 67.72% inhibition, while at the lowest dose given the percentage inhibition was 17% (Fig. 3A). GST activity levels were studied at different concentration of the extracts of *C. chinensis*. Here, we showed a dose response activity of GST with the highest activity of 54.24 µmole CDNB conjugated/min/mg protein at concentration of 12.5 µg/µl dose (Fig. 3B). To investigate the free radical scavenging activities, we examined the DPPH and ABTS free radical scavenging activity of *C. chinensis* extracts. The DPPH free radical scavenging activity of the extracts revealed that inhibition was dose dependent, and the maximum inhibition (83.17%) was observed in the highest concentration of 12.5 µg/µl (Fig. 3C). For the standard ascorbic acid, the concentration tested was 10 times diluted as those of the respective extract concentrations, that means at 1.25 µg/µl standard solution the percentage scavenging activity was 55.6 (Fig. 3D). Hydroalcoholic extract of *C. chinensis* showed dose dependent increase in ABTS scavenging activity with the maximum inhibition as high as 95.21% in the highest dose of 12.5 µg/µl (Fig. 3E). A comparison was made between the hydroalcoholic extract, and the standard (Trolox) treated with ten-fold decrease in the concentration as that of the extract. The percentage scavenging activity of ABTS standard (Trolox) was found to be 50.14% at the maximum dose of 1.25 µg/µl (Fig. 3F).

### In-vitro anti-inflammatory bioassay (Protein denaturation assay)

The protein denaturation bioassay (in-vitro assessment) of hydro-alcoholic extract of *C. chinensis* was analyzed and the results are depicted in Table 3. It revealed that hydro-alcoholic extract of *C. chinensis* inhibited protein denaturation in a concentration-dependent manner (1600 µg to 2200 µg/ml) as increased absorbance was observed. IC_50_ value of the crude extract was observed to be 1592.308 µg/ml as against 74.94 µg/ml of the positive control diclofenac (Table 3), thus depicting its anti-inflammatory activity.

**Table 3:** Effect of hydro-alcoholic extract of *C. chinensis* and standard drug (diclofenac) on protein denaturation.

Concentration(µg/ml)	Percentage inhibition (%)	IC_50_ Value
*C. chinensis* Extract		
1000	20.99 ± 0.36	1592.308
1400	32.39 ± 2.20	
1800	69.28 ± 0.82	
2200	72.88 ± 1.44	
2600	81.28 ± 2.42	
3000	94.17 ± 0.11	
Standard drug (diclofenac)		
20	6.94 ± 1.05	74.94
40	26.69 ± 0.72	
60	40.27 ± 0.92	
80	53.30 ± 0.43	
100	67.05 ± 0.48	
150	94.69 ± 0.04	
200	95.12 ± 0.26	

### Volatile compounds of *C. chinensis* by GCMS

**Figure 4:**
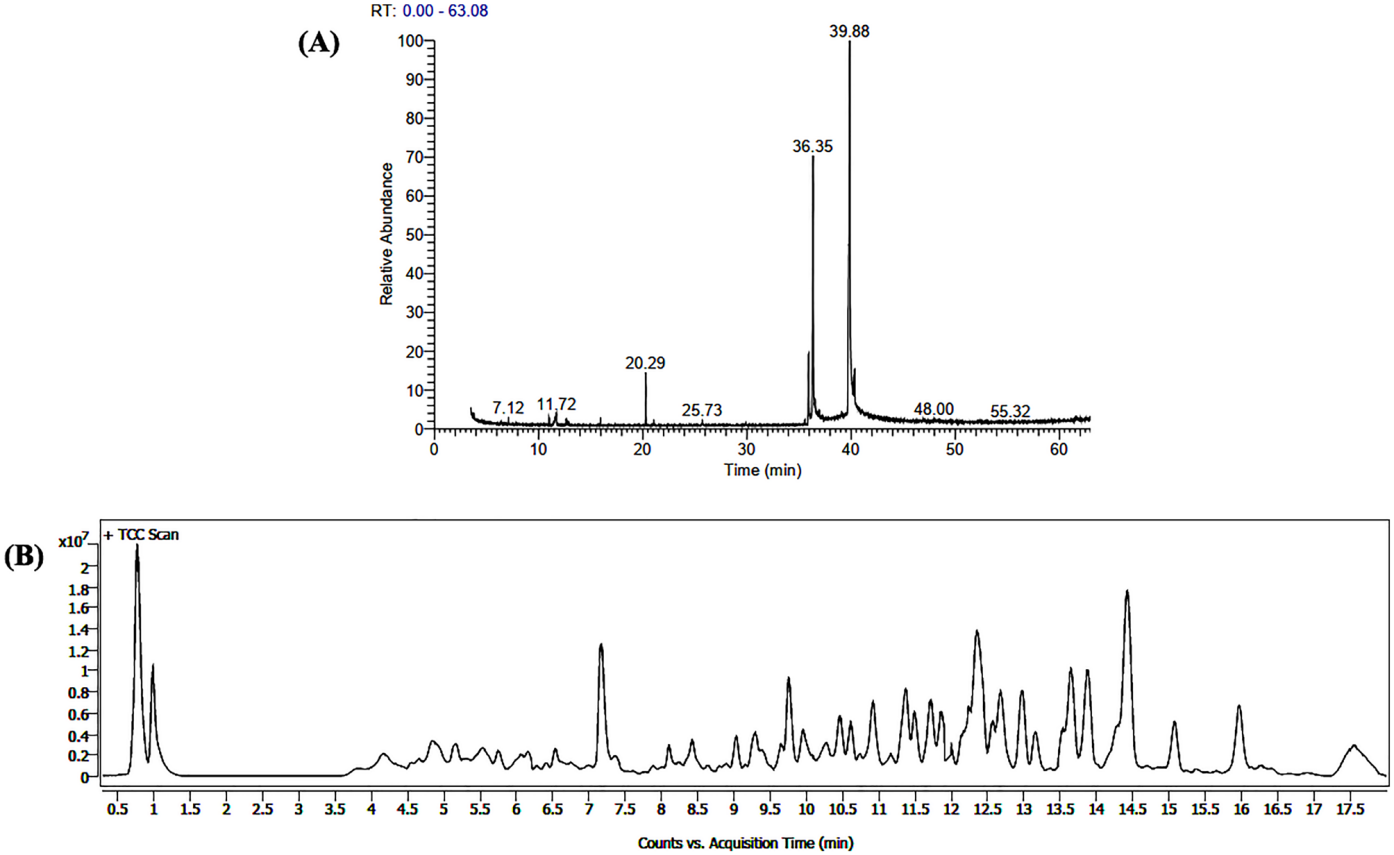
(**A**) Represents the GCMS chromatograph of *C.chinenis.* (**B**) Represents the LCMS chromatograph of *C.chinensis.*

The GCMS analysis was performed to determine the presence of important compounds in the hydro-alcoholic extract of *C. chinenis*. A total of 61 compounds were identified (Fig. 4A) in which the major constituent was (1S,15S)-Bicyclo [13.1.0] hexadecan-2-one (ketone) (50.48%), 2,6,10,14-Tetramethylpentadecan-6-ol,9(E) (alcohol) (26.24%), (E)-9-Octadecenoic acid ethyl ester (ester) (2.03%) and 11(E)-Conjugated linoleic acid, ethyl ester (ester) (1.34%) (Table 4).

**Table 4:** Chemical composition of *Coridius chinensis* hydro-alcoholic extract.

Sl.no	RT^a^	Components	RSI^b^	%RA^C^
1	4.60	Cyclobutene, 2-propenylidene	919	0.08
2	5.11	Ethyne, fluoro-	929	0.05
3	5.35	2,2,3,3,4,4-Hexamethyltetrahydrofuran	771	0.06
4	6.41	2-Pentanone, 4-hydroxy-4-methyl-	855	0.16
5	6.89	Benzene, [(methylsulfinyl)methyl]-	932	0.05
6	7.12	Benzene, 1,3-dimethyl-	916	0.35
7	7.79	p-Xylene	836	0.04
8	10.31	1,2-Pentadiene, 4-methoxy-4-methyl-	793	0.05
9	11.71	2-Hexenoic acid, (E)-	932	1.83
10	12.67	Benzene, 1,4-diethyl-	887	0.36
11	12.86	o-Cymene	835	0.23
12	13.06	Benzoic acid, 3-methyl-, 2-oxo-2-phenylethyl ester	875	0.03
13	15.96	2-Hydroxymandelic acid, ethyl ester, di-TMS	861	0.43
14	16.93	1H-Indene, 1-methylene-	894	0.03
15	19.20	2-Decenal, (E)-	905	0.07
16	20.29	Heptane, 3,3-dimethyl-	905	3.04
17	24.45	Pyridine, 3-(1a,2,7,7a-tetrahydro-2-methoxy-1-phenyl-1,2,7-metheno-1H-cyclopropa[b]naphthalen-8 -yl)-	914	0.05
18	27.34	1,3-Benzenediol, 4,6-dichloro-2-methyl-	795	0.04
19	28.22	3-Ethyl-7-hydroxyphthalide	860	0.08
20	28.46	3-Pentenoic acid, 2,2,4-trimethyl-	867	0.04
21	29.03	Cyclotridecane	901	0.07
22	29.68	2H-Azepin-2-one, hexahydro-7-methyl-	729	0.04
23	30.66	Amphetamine-3-methyl	903	0.07
24	30.99	Phenol, 4-[2-(methylamino)ethyl]-	919	0.04
25	31.10	L-Alanine, ethyl ester	877	0.04
26	31.94	Stannane, trimethyl[[(trifluoromethyl)sulfinyl]oxy]-	808	0.06
27	34.46	Phthalic acid, butyl hept-4-ylester	893	0.04
28	35.17	1,5-Diazacycloheptadecan-6-one, 1-acetyl	800	0.07
29	35.58	Tetradecanoic acid, 10,13-dimethyl-, methyl ester	898	0.3
30	35.93	Palmitoleic acid	915	5.8
31	36.35	2,6,10,14-Tetramethylpentadecan-6-ol	898	26.24
32	36.57	1-Hexyl-2-nitrocyclohexane	983	0.57
33	36.74	9,9-Dimethoxybicyclo[3.3.1]nona-2,4-dione	849	0.11
34	36.96	Undecanoic acid, 2,8-dimethyl-, methyl ester	850	0.4
35	37.53	Dihydroedulan IIA	878	0.03
36	38.02	9,9-Dimethoxybicyclo[3.3.1]nona-2,4-dione	855	0.04
37	38.33	Propanamide, N-(aminocarbonyl)-	960	0.06
38	38.99	Dihydroedulan IIA	879	0.06
39	39.09	Methyl (Z)-10-pentadecenoate	879	0.29
40	39.56	Dimethyl 2-carbethoxy-cyclopropane-1,1-dicarboxylate	828	0.04
41	39.88	(1S,15S)-Bicyclo[13.1.0]hexadecan-2-one	899	50.48
42	40.25	9(E),11(E)-Conjugated linoleic acid, ethyl ester	915	1.34
43	40.35	E)-9-Octadecenoic acid ethylester	900	2.03
44	40.81	5,10-Pentadecadienoic acid,(E,E)-	941	0.07
45	41.08	Spiro[3.5]nonan-1-one, 5-methyl-, trans-	810	0.04
46	45.17	1,2,4-Benzenetricarboxylic acid, 1,2-dimethyl nonyl ester	673	0.05
47	46.20	4-Trimethylsilylmethyl-6-(2-phenylethyl)-5,6-dihydro-2H-pyran	733	0.08
48	46.52	Ethyl 4,4-dimethyl-5-oxo-tetrahydro furan-3-carboxylate	819	0.09
49	46.93	Methyl 4-oxodecanoate	834	0.26
50	48.00	Phthalic acid, di(2-propylpentyl) ester	884	0.48
51	48.46	4,4-Dipropylheptane	808	0.09
52	49.49	3-Ethyl-2,6,10-trimethylundecane	838	0.22
53	52.23	5-Decene, 1-bromo-, (Z)-	825	0.21
54	52.42	6-Tetradecanesulfonic acid, butyl ester	766	0.1
55	53.01	1,2-Dicarboxy-3-(4-chlorophenyl)-2,3(1H)-dihydropyrido(1, 2-a)benzimidazole	836	0.04
56	54.24	1-Hydroxycyclohexanecarboxylic acid	797	0.05
57	55.29	3-Isopropyl-6,10-dimethylundecane-2-ol	831	0.04
58	55.34	3-Methyltetracosane	753	0.06
59	59.14	2-Isopropyl-6-phenylnicotinonitrile	749	0.07
60	61.65	Decane, 3-bromo-	884	0.43
61	62.28	4-Methyl-2,4-bis(p-hydroxyphenyl)pent-1-ene, 2TMS derivative	766	0.04

Total Compounds Identified (%) 97.71.

^a^Retention time.

^b^Reverse Similarity index on TG-5MS capillary column.

^c^Relative area (Peak area relative to the total peak area).

### Compound profiling of *C. chinensis* by LCMS

LCMS analysis of the hydro-alcoholic extract of *C. chinenis* detected a total of 829 compounds of which 27 compounds were found to be biologically active (Fig. 4B). Compounds such as Morphine 3-glucuronide, Ecgonine, Ecgonine methyl ester, Sufentanil, Palmitoyl Ethanolamide, Etodolac glucuronide, etc. were detected through LC–MS analysis of the hydroalcoholic extract of *C. chinensis* (Table 5).

**Table 5:** List of bioactive and therapeutic compounds detected by LC–MS from hydro-alcoholic extract of *Coridius chinensis*.

Compound name	Formula	Mass	RT	Score	Properties^41^
Morphine 3-glucuronide	C_23_ H_27_ N O_9_	462.1757	10.926	94.99	Opiate, analgesic, metabolite or morphine
Palmitoyl Ethanolamide	C_18_ H_37_ N O_2_	322.2703	12.798	94.05	Anti-Inflamatory agent, Antiviral agent
Edrophonium	C_10_ H_16_ N O	166.1225	0.789	88.05	Anticholinesterase drug
Enalaprilat	C_18_ H_24_ N_2_ O_5_	348.1673	5.638	87.87	Orally active angiotensin-converting enzyme inhibitor. Does not contain a sulfhydryl group. metabolite of Enalapril
Minoxidil	C_9_ H_15_ N_5_ O	209.1278	0.996	86.6	Treatment of hypertension (vessel dilator)
Atenolol	C_14_ H_22_ N_2_ O_3_	266.1634	12.528	86.17	B1-selective andrenergic receptor antagonist
Zidovudine	C_10_ H_13_ N_5_ O_4_	267.0961	0.994	96.72	Treatment of HIV
Neamine (Neomycin A)	C_12_ H_26_ N_4_ O_6_	322.1858	6.061	96.45	Antibiotic Drug, component of Neomycin
Gabapentin	C_9_ H_17_ N O_2_	171.125	10.526	85.99	Structural analog of GABA an important inhibitory neurotransmitter
Succinylcholine	C_14_ H_30_ N_2_ O_4_	290.2207	11.795	84.56	Short-acting skeletal muscle relaxant
Ecgonine methyl ester	C_10_ H_17_ N O_3_	199.12	5.566	83.94	Topical local anesthetic, relieves pain in cacer patients, stimulant effect on the CNS, inactivation of epinephrine and norepinephrine and blockade of norepinephrine uptake, metabolite cocaine
Ecgonine	C_9_ H_15_ N O_3_	185.1044	3.899	83.88	Topical local anesthetic, relieves pain in cacer patients, stimulant effect on the CNS, inactivation of epinephrine and norepinephrine and blockade of norepinephrine uptake, metabolite cocaine
Penciclovir	C_10_ H_15_ N_5_ O_3_	253.1168	5.645	83.43	Antiviral prodrug active against herpes simplex 1(HSV-1) and 2 (HSV-2) and varicella zoster (VSV)
Diglykokoll	C_4_ H_7_ N O_4_	133.037	0.802	83.38	Pharmacological Action: Chelating Agents
Methoxamine	C_11_ H_17_ N _O3_	211.1199	5.351	83.14	Treatment of hypotension
Selegiline	C_13_ H_17_ N	187.1353	4.788	82.49	Selective inhibitor of B-type monoamine oxidase, Treatment of Parkinsons Disease
Allopurinol	C_5_ H_4_ N_4_ O	136.0381	4.810	82.35	Ability to inhibit the synthesis of uric acid
Methylphenidate	C_14_ H_19_ N O_2_	233.1407	7.047	81.22	Treatment of ADD(ADHD) and narcolepsy
Metoprolol	C_15_ H_25_ N O_3_	267.1822	8.728	81.09	Treatment of Hypertension and varied heart complications
Tubocurarine	C_37_ H_41_ N_2_ O_6_	609.2994	11.497	81.02	Non-depolarizing neuromuscular blocking drug
Acetylcarnitine	C_9_ H_18_ N O_4_	204.1229	1.004	80.78	Treatment of carnitine deficiency; Natural aminoacid Endogenous Metabolite of carnitine
Dihydrostreptomycin	C_21_ H_41_ N_7_ O_12_	583.2836	10.829	79.81	Antibiotic, Treatment of tuberculosis; Metabolite of Streptomycin
Oleoyl Ethanolamide	C_20_ H_39_ N O_2_	325.2967	13.5	79.62	Pharmacological Action: Enzyme Inhibitors
Sufentanil	C_22_ H_30_ N_2_ O_2_ S	386.2031	5.908	79.43	Most potent opioid available, anesthetic
Dicyclomine	C_19_ H_35_ N O_2_	309.2655	13.936	79.25	Anticholinergic; Antispasmodic
Etodolac glucuronide	C_23_ H_29_ N O_9_	463.1843	9.309	77.92	Non-steroidal anti-inflammatory. metabolite of Etodolac
Nimodipine	C_21_ H_26_ N_2_ O_7_	418.1746	12.340	77.46	Calcium slow channel antagonist (varied applications including treatment of subarachnoid hemorrhage)
Morphine 3-glucuronide	C_23_ H_27_ N O_9_	462.1757	10.926	94.99	Opiate, analgesic, metabolite or morphine
Palmitoyl Ethanolamide	C_18_ H_37_ N O_2_	322.2703	12.798	94.05	Anti-Inflamatory agent, Antiviral agent
Edrophonium	C_10_ H_16_ N O	166.1225	0.789	88.05	Anticholinesterase drug
Enalaprilat	C_18_ H_24_ N_2_ O_5_	348.1673	5.638	87.87	Orally active angiotensin-converting enzyme inhibitor. Does not contain a sulfhydryl group. metabolite of Enalapril
Minoxidil	C_9_ H_15_ N_5_ O	209.1278	0.996	86.6	Treatment of hypertension (vessel dilator)
Atenolol	C_14_ H_22_ N_2_ O_3_	266.1634	12.528	86.17	B1-selective andrenergic receptor antagonist
Zidovudine	C_10_ H_13_ N_5_ O_4_	267.0961	0.994	96.72	Treatment of HIV
Neamine (Neomycin A)	C_12_ H_26_ N_4_ O_6_	322.1858	6.061	96.45	Antibiotic Drug, component of Neomycin
Gabapentin	C_9_ H_17_ N O_2_	171.125	10.526	85.99	Structural analog of GABA an important inhibitory neurotransmitter
Succinylcholine	C_14_ H_30_ N_2_ O_4_	290.2207	11.795	84.56	Short-acting skeletal muscle relaxant
Ecgonine methyl ester	C_10_ H_17_ N O_3_	199.12	5.566	83.94	Topical local anesthetic, relieves pain in cacer patients, stimulant effect on the CNS, inactivation of epinephrine and norepinephrine and blockade of norepinephrine uptake, metabolite cocaine
Ecgonine	C_9_ H_15_ N O_3_	185.1044	3.899	83.88	Topical local anesthetic, relieves pain in cacer patients, stimulant effect on the CNS, inactivation of epinephrine and norepinephrine and blockade of norepinephrine uptake, metabolite cocaine
Penciclovir	C_10_ H_15_ N_5_ O_3_	253.1168	5.645	83.43	Antiviral prodrug active against herpes simplex 1(HSV-1) and 2 (HSV-2) and varicella zoster (VSV)
Diglykokoll	C_4_ H_7_ N O_4_	133.037	0.802	83.38	Pharmacological Action: Chelating Agents
Methoxamine	C_11_ H_17_ N O_3_	211.1199	5.351	83.14	Treatment of hypotension
Selegiline	C_13_ H_17_ N	187.1353	4.788	82.49	Selective inhibitor of B-type monoamine oxidase, Treatment of Parkinsons Disease
Allopurinol	C_5_ H_4_ N_4_ O	136.0381	4.810	82.35	Ability to inhibit the synthesis of uric acid
Methylphenidate	C_14_ H_19_ N O_2_	233.1407	7.047	81.22	Treatment of ADD(ADHD) and narcolepsy
Metoprolol	C_15_ H_25_ N O_3_	267.1822	8.728	81.09	Treatment of Hypertension and varied heart complications
Tubocurarine	C_37_ H_41_ N_2_ O_6_	609.2994	11.497	81.02	Non-depolarizing neuromuscular blocking drug
Acetylcarnitine	C_9_ H_18_ N O_4_	204.1229	1.004	80.78	Treatment of carnitine deficiency; Natural aminoacid; Endogenous Metabolite of carnitine
Dihydrostreptomycin	C_21_ H_41_ N_7_ O_12_	583.2836	10.829	79.81	Antibiotic,Treatment of tuberculosis: Metabolite of Streptomycin
Oleoyl Ethanolamide	C_20_ H_39_ N O_2_	325.2967	13.5	79.62	Pharmacological Action: Enzyme Inhibitors
Sufentanil	C_22_ H_30_ N_2_ O_2_ S	386.2031	5.908	79.43	Most potent opioid available, anesthetic
Dicyclomine	C_19_ H_35_ N O_2_	309.2655	13.936	79.25	Anticholinergic; Antispasmodic;
Etodolac glucuronide	C_23_ H_29_ N O_9_	463.1843	9.309	77.92	Non-steroidal anti-inflammatory. metabolite of Etodolac
Nimodipine	C_21_ H_26_ N_2_ O_7_	418.1746	12.340	77.46	Calcium slow channel antagonist (varied applications including treatment of subarachnoid hemorrhage)

Reference: ^41^.

## Discussion

People around the world are using insects as food and for therapeutic purposes. A very limited study was reported on the composition and its therapeutic validation on *C. chinensis*. The present study was designed to evaluate the underlying medicinal benefit and to extrapolate possible health implications and pharmacological benefits from *C. chinensis*. Our study revealed the presence of nutritional and medicinally dynamic constituents in the different extracts of *C. chinensis.*

Edible insects contain various metabolites. There are reports on the presence of various primary and secondary metabolites like steroids, triterpenoids, cardiac glycosides, anthraquinones, flavonoids, tannins, alkaloids, amino acids and reducing sugars in methanolic extract of *Henicus whellani* (crickets) and *Macrotermes facilger* (termites)^7^. Flores et al. (2020)^26^ reported the presence of proteins, reducing sugars and saponins in the aqueous extract of two edible insects, viz. *Tenebrio molitor* and *Ulomoides dermestoides*. Saponins are amphiphilic in nature, therefore they are soluble in both non-polar as well as polar solvent^27^. *Henicus whellani* and *Macrotermeds falciger* showed higher amounts of phenols and flavonoids than the *C. chinensis*^7^. The presence of some secondary metabolites like tannins flavonoids, and alkaloids are known to show medicinal activity and commonly associated with antioxidant activity and anti-inflammatory^28,29^. In our study, we reported the presence of secondary metabolites such as alkaloid, terpenoids, cardiac glycosides, saponins and flavonoids, and primary metabolites like proteins and reducing sugars in the hydro-alcoholic extract. Hexane extract has only reducing sugars and saponins. The presence of several secondary metabolites in the hydro-alcoholic extract could have significant impact on the antioxidant and anti-inflammatory activity of *C. chinensis.* Insect stability and vulnerability to microbial infection are often gauged by the moisture content of the insects. *C. chinensis* has a moisture content of 50.46% which when compared to other aquatic insects like *Crocothemis servilia, Cybister tripanctatus, Hydrophilus olavaceous, Lacotrephes maculatus, Lethocerus indicus* found similar or slightly higher. It is also known to us that insects are rich in nutrients such as proteins, fats, carbohydrates, and vitamins^30^. Due to different food habits or different species, the composition content of insect varies. The insects have high protein content and appreciably it will lead to total protein intake of indigenous populations. Generally, edible insects contain proteins in the range of 35%–60% or 10%–25% on dry weight and fresh weight basis respectively^31^. Our study revealed that the protein content of *C. chinensis* on fresh weight was 22.12% which fit the general range. While fat is the second largest component of insect nutrient^32^, Rumpold and Schluter (2013)^30^ reported that many of the insect orders such as Orthoptera, Lepidoptera, Blattodea, Isoptera, Hemiptera, and Coleoptera have the fat content of 13.41%, 27.66%, 29.90%, 32.74%, 30.26%, and 33.40%, respectively. The fat contents of *C. chinensis* was relatively lower than the average fat content of Hemiptera. Similarly, the carbohydrate content was reported to be 2.2% which is comparatively lower than the other previously reported ranges i.e., 6.71% (stink bug) to 15.98% (cicada)^32^. The presence of biologically active principles, that is, flavonoids, and phenolic compounds, suggested either of the constituents or synergistically is responsible for producing the analgesic and anti-inflammatory effects^33^. Insect’s antioxidant enzyme system plays a vital role in maintaining the homeostasis of many important metabolisms. Dutta et al. (2016)^34^ studied the superoxide radical scavenging activities of aqueous extract of *Vespa affinis* L. (AEVA) and found that the maximum inhibitory activity of superoxide radicals was observed at a dose of 10 µg/µl. Insect’s antioxidant enzyme system also contains reduced glutathione (GSH), which helps in reducing the reactive oxygen species (ROS) through the formation of oxidized glutathione (GSSH). Our study revealed that the hydro-alcoholic extract of *C. chinensis* has significant antioxidant properties as shown in Fig. 3. DPPH free radical scavenging activity of *C. chinensis* was also compared to the standard ascorbic acid with ten-fold dilution. It was indicative that the hydro-alcoholic extract has significant scavenging activity. *Coridius nepalensis*, a close relative of *C. chinensis,* at 10 mg/ml. Aqueous extract of *C. nepalensis* showed 65.16% DPPH radical scavenging and 100 µg/ml ascorbic acid showed 46.97%^12^. However, other studies reported that methanol-soluble extracts of all the insects analyzed showed strong activities of DPPH free radical scavenging activity. Comparatively among the order, Lepidopterans (mulberry silkworms) with IC_50_ value 18.4 ± 0.09 showed the highest DPPH radical scavenging activity as compared to IC_50_ value of Hymenopteran (weaver ant adult) (84.92 ± 0.19), Hemipteran (stink bug) (164.90 ± 0.12), and Orthopteran (cricket) (126.34 ± 0.17)^35^.

ABTS is a greatly sensitive method of identifying antioxidant activity because of its faster reaction kinetics and a sharp response to antioxidant^36^. ABTS when react with potassium persulfate form ABTS radical cation (ABTS• +). Addition of an antioxidant to a fairly stable ABTS• + will instantaneously react with the free radical and thereby scavenging it. The percentage inhibition of ABTS radical cation depends on the concentration of antioxidants as well as the duration of the reaction^23^. Comparison between the hydro-alcoholic extracts and standard trolox with respect to the percentage inhibition of ABTS radical cation is shown in Fig. 3E,F. Mohsin et al. (2020)^35^ reported that ABTS scavenging activity (IC_50_: μg/ml) was higher in termite species.

It is a novel idea that anti-inflammatory properties of edible insects should be investigated and the mechanism underlying the anti-inflammatory potential of insect needs to be clarified. Anti-inflammatory activity is demonstrated by an increase in inhibition of protein (albumin) denaturation or increase in protein stability due to the extract exposure. This has been shown by a reasonable level of IC_50_ value (1592.308 µg/ml) in *C. chinensis.*

GCMS analysis demonstrated the presence of ketone, alcohol and ester in *C. chinensis* which have important value in food and therapeutic purposes. The conjugated linoleic acid is an important compound which has anti-cancer property, prevents heart disease, improves immune function, and alters body composition to treat obesity^37^. Other major compounds like E)-9-Octadecenoic acid ethyl ester compound were reported to have perfumery applications. Recently, *C. chinensis* has been given a lot of attention for the isolation of natural compounds. A study has reported that the methanolic extract of *C. chinensis* contains oleic acid and palmitic acid as their main compounds^38^. The methanolic extract of *Encosternum delegorguei* (stink bug) contains 18 compounds which were aliphatic hydrocarbons, aldehydes, furanones, aromatic, oxo-alkenals, esters, ketones, lactones and ethers^39^. The results supported the traditional use of these edible insects in the treatment of pain conditions. The synthesized peptides from insect’s *(Gryllodes sigillatus, Tenebrio molitor, Schistocerca gragaria*) protein showed anti-inflammatory activity^29^.

Among the many compounds detected in LCMS, the compounds Morphine 3-glucuronide, Ecgonine, Ecgonine methyl ester and Sufentanil are opiate analgesic and topical local anaesthetic. This corroborates with the traditional practice of using the insect in relieving pain. LCMS detection of the compounds Palmitoyl Ethanolamide and Etodolac glucuronide in *C. chinensis* supports our finding of *in-vitro* anti-inflammatory assay. Palmitoyl Ethanolamide is a well-documented anti-inflammatory, analgesic, immunomodulatory, antimicrobial and neuroprotective compound with no side effects in man^40^. Etodolac glucuronide has anti-inflammatory effects at higher dose and analgesic activity at lower dose^41,42^. A low to moderate level of these two compounds in *C. chinensis* is likely responsible for the traditional entomotherapeutic use for anti-inflammatory and analgesic effects. The study has thus been able to scientifically validate the traditional entomotherapeutic practice of using *C. chinensis*.

## Conclusion

The present study supports that *C. chinensis* is an alternate food resource with several health benefits to human. Further in-depth investigation can reveal many of its rich pharmaceutical applications. *C. chinensis* is rich in various primary and secondary metabolites with numerous therapeutic properties that can be translated into nutraceuticals, medicine, food supplement and other industrial applications. Sustainable commercial-scale production of *C. chinensis* to supplement nutritional and therapeutic requirements can be exploited as a future prospect.

## Data Availability

All data generated or analyzed during current study are available in the manuscript, figures and tables.
